# Etiology and Risk Factors for Infectious Keratitis in South Texas

**DOI:** 10.18502/jovr.v15i2.6729

**Published:** 2020-04-06

**Authors:** Madeleine Puig, Menachem Weiss, Ricardo Salinas, Daniel A Johnson, Ahmad Kheirkhah

**Affiliations:** Department of Ophthalmology, Long School of Medicine, University of Texas Health at San Antonio, San Antonio, Texas, USA

**Keywords:** Acanthamoeba, Bacteria, Corneal Ulcer, Fungus, Keratitis

## Abstract

**Purpose:**

To determine the causative organisms and associated risk factors for infectious keratitis in South Texas.

**Methods:**

This retrospective study was performed at a tertiary teaching hospital system in South Texas. Medical records of all patients who presented with infectious keratitis from 2012 to 2018 were reviewed. Only patients with culture-proven bacterial, fungal, and *Acanthamoeba* keratitis were included.

**Results:**

In total, 182 eyes of 181 patients had culture-proven bacterial, fungal, or *Acanthamoeba* keratitis. The age of patients ranged from 3 to 93 years, with a mean of 48.3 ± 20.8 years. The most common etiologic agent was bacteria, with 173 bacterial cultures (95.1%) recovered, followed by 13 fungal cultures (7.1%), and 3 *Acanthamoeba* cultures (1.6%). Of the 218 bacterial isolates, coagulase-negative *Staphylococcus* was the most common (25.7%), followed by *Pseudomonas aeruginosa* (23.4%), *Staphylococcus aureus* (11.0%), and *Moraxella* (7.8%). *Fusarium* was the most common fungal isolate (46.2%). The most common risk factors for infectious keratitis included contact lens wear (32.4%), underlying corneal disease (17.6%), trauma (14.3%), and ocular surface disease (13.7%).

**Conclusions:**

Bacteria are the most common cause of infectious keratitis in this patient population, with coagulase-negative *Staphylococcus* and *Pseudomonas* as the most common isolates. The prevalence of culture-positive fungal keratitis is significantly lower than that of bacterial keratitis. Contact lens wear is the most common risk factor associated with infectious keratitis in South Texas.

##  INTRODUCTION

Infectious keratitis is a vision-threatening infection of the cornea caused by bacteria, fungi, parasites, or viruses. Infection usually begins with epithelial defects in the setting of a weakened ocular defense system and proceeds to stromal invasion, necrosis, and corneal ulceration.^[1]^ Ulcers can lead to visual impairment and may lead to corneal perforation and endophthalmitis. Outcomes of these patients depend on timely diagnosis and treatment with close follow-up.

In 2010 alone, keratitis accounted for over 700,000 doctor visits in the United States, 76.5% of which led to antimicrobial prescriptions. The combined cost of these visits is estimated to be $175 million in direct healthcare expenditure.^[2]^

Clinical management of infectious keratitis depends on the etiology of the infection, the extension of corneal involvement, practice location, risk factors, and response to previous therapy.^[3,4,5]^ Although the success of initial therapy relies on empiric broad spectrum antibiotics, identifying the organism responsible for the infection is valuable for a more specific treatment. Stains of smears obtained from the ulcer may help determine the etiologic agent; however, cultures are ideal for diagnosing infectious keratitis, isolating the organism, and performing antibiotic sensitivity. Antibiotic selection may be influenced by the availability of the drugs, cost of the treatment, and spectrum of pathogens in the community.^[6,7]^ Therefore, efforts have been made to characterize geographic and temporal spectrums of responsible pathogens as well as antimicrobial resistance in different populations.^[5,8,9,10,11,12]^ Bacterial infections predominate over other etiologies, with Gram-positive bacteria being more common than Gram-negative ones.^[12]^ Fungal infections are responsible for a greater percentage of infectious keratitis in tropical climates, during summer months, and among agricultural workers.^[12,13,14,15,16]^ *Acanthamoeba* keratitis is rare but is typically associated with contact lens wear.^[1]^

The purpose of this study was to characterize the etiologies of infectious keratitis presenting to a tertiary teaching hospital system in South Texas to better guide future treatment. We aimed to identify the microbial spectrum in this population and compare these data to reports from other parts of the country as well as around the world. We hypothesized that the etiology and microbial spectrum of infectious corneal ulcers in South Texas would be comparable to those in other locations with similar temperate climates, such as Dallas (Texas) and Los Angeles (California), and would vary from those in tropical climates, such as Florida.

##  METHODS

This is a retrospective chart review study of patients visited at the University Hospital System as well as the University of Texas Health at San Antonio (San Antonio, Texas), both of which provide tertiary ophthalmic care to South Texas. The study protocol was approved by the Institutional Review Board at the University of Texas Health at San Antonio, and the study complied with the Health Insurance Portability and Accountability Act (HIPAA).

Patients were identified by screening electronic medical records for encounters with infectious keratitis between 2012 and 2018. The screen included multiple International Classification of Diseases (ICD)9 and ICD10 codes to identify patients with keratitis and corneal ulcers. This search yielded 622 patient charts, which were each individually reviewed to confirm a diagnosis of bacterial, fungal, or *Acanthamoeba* keratitis proven by a positive culture result. Eyes with viral keratitis, negative cultures, or those which were not cultured were excluded.

Smears and cultures were obtained on a case-by-case basis but were typically collected in cases with infiltrates that were central, large, deep, chronic, refractory to antibiotic treatment, or atypical. Corneal scrapings from the ulcer were directly obtained with a sterile cotton swab or sterile calcium alginate swab and immediately inoculated onto blood agar, chocolate agar, enriched thioglycolate broth, Page's saline, universal transport media, viral broth, and Sabouraud dextrose agar. In addition, the scrapings were also placed on slides for Gram staining and potassium hydroxide wet mounts. Inoculated plates were immediately taken to the laboratory for microbiologic evaluation.

From each chart, demographic and clinical data were collected including age, sex, culture isolate, and risk factors such as, but not limited to, contact lens wear, preceding trauma or inciting event, ocular disease, immunodeficiency, and steroid use.

##  RESULTS

Of the 621 patients with presumptive infectious keratitis visited between 2012 and 2018, there were a total of 182 eyes of 181 patients with culture-proven bacterial, fungal, or *Acanthamoeba* keratitis. A total of 440 eyes were excluded because cultures were not obtained (*n* = 279) or were negative (*n* = 157), or the corneal ulcers were proven by laboratory studies to be secondary to a viral infection (*n* = 4). Therefore, of the eyes for which cultures were obtained, 53.6% had a positive culture.

Of the 181 patients, 92 were female and 89 were male. Corneal ulcers were in the right eye in 106 and in the left eye in 74 patients, and were bilateral in one patient. The mean patients' age was 48.3 ± 20.8 years (range, 3–93 years); 11 patients (6.1%) were younger than 18 years old, 53 (29.3%) were between 18 and 40 years old, and 117 patients (64.6%) were older than 40 years old; 103 (56.9%) patients were identified as Hispanic, 61 (33.7%) as Caucasian, 8 (4.4%) as African American, 3 (1.7%) as Asian, and 6 (3.3%) as other ethnicities.

Of the total 182 eyes, cultures identified bacteria in 173 eyes (95.1%), fungi in 13 eyes (7.1%), and *Acanthamoeba* in 3 eyes (1.6%); 146 eyes (80.2%) had monomicrobial infection and 36 eyes (19.7%) had polymicrobial infection. All three cases of *Acanthamoeba *and four cases of fungal keratitis occurred in patients with concurrent bacterial ulcers.

### Bacterial Keratitis

Table 1 demonstrates all 218 bacterial organisms isolated during the study period. Of the 36 eyes with polymicrobial infections, 27 eyes demonstrated growth of two organisms, and 9 eyes revealed three or more organisms. The most common bacterial isolate was coagulase-negative *Staphylococcus *(CoNS) (*n* = 56, 25.7%), followed by *Pseudomona*s *aeruginosa* (*n* = 51, 23.4%), and *Staphylococcus aureus* (*n* = 24, 11.0%). In addition, 17 isolates (7.8%) of *Moraxella* were identified.

**Table 1:** Spectrum of bacterial isolates observed in cases with bacterial keratitis in South Texas


**Bacteria**	**Number of cases**	**Percentage of Bacterial Isolates**
*Coagulase-negative Staphylococcus*	56	25.7%
*Pseudomonas aeruginosa*	51	23.4%
*Staphylococcus aureus*	24	11.0%
*Moraxella species*	17	7.8%
*Propionibacterium acnes*	9	4.1%
*Streptococcus viridans*	8	3.7%
*Diphtheroids*	8	3.7%
*Bacillus species*	6	2.8%
*Streptococcus pneumoniae*	6	2.8%
*Serratia marcescens*	5	2.3%
*Methicillin-resistant Staphylococcus aureus*	4	1.8%
*Actinobacter*	3	1.4%
*Achromobacter*	2	0.9%
*Enterobacter species*	2	0.9%
*Enterococcus species*	2	0.9%
*Streptococcus anginosus*	2	0.9%
*Rothia species*	1	0.5%
*Abiotrophia*	1	0.5%
*Actinomyces meyeri*	1	0.5%
*Atypical mycobacteria (mycobacterium chelonae-abscessus complex)*	1	0.5%
*Capnocytophaga species*	1	0.5%
*Corynebacterium species*	1	0.5%
*Granulicatella species*	1	0.5%
*Group B Streptococcus*	1	0.5%
*Group G Streptococcus*	1	0.5%
*Haemophilus parainfluenzae*	1	0.5%
*Morganella*	1	0.5%
*Neisseria species (not meningitidis or gonorrhea)*	1	0.5%
*Stenotrophomonas Maltophilia*	1	0.5%
Total	218	

**Table 2:** Predisposing risk factors identified in patients with culture-proven keratitis based on etiology


	**Bacterial (** ***n*** ** = 172)**	**Fungal (** ***n*** ** = 13)**	*Acanthamoeba * **(** ***n*** ** = 3)**
Contact lens wear	57	2	2
Underlying corneal disease	29	3	0
Trauma	24	2	0
Ocular surface disease	25	1	0
Risk factors not addressed in documentation	16	3	1
Immunocompromised only	10	0	0
Topical steroid use	6	0	0
Recent history of ophthalmic procedure	3	2	0
Exposure to contaminated water	2	0	0

**Table 3:** Predisposing factors identified in patients with corneal ulcer caused by the most prevalent bacterial isolates


	**CoNS**	*P. aeruginosa*	*S. aureus*	*Moraxella spp. *	*P. acnes*	*S. viridans*	*Diphtheroids*	*S. pneumoniae*	*Bacillus spp. *	*S. marcescens*	**MRSA**
Contact lens wear	16	33	4	2	2	1	0	1	0	4	0
Trauma	5	6	4	5	3	2	0	1	2	0	1
Ocular surface disease	13	3	2	1	1	1	3	0	1	1	0
Underlying corneal disease	13	3	5	3	1	1	6	1	2	0	0
Exposure to contaminated water	0	1	0	0	2	0	0	0	0	0	0
Prior viral keratoconjunctivitis	1	0	0	1	0	0	0	0	0	0	0
Recent history of ophthalmic procedure	0	0	2	1	0	0	0	0	0	0	0
Topical steroid use	2	0	1	0	0	0	0	0	1	0	1
Immunocompromised only	2	2	4	2	0	0	0	2	0	0	1
Risk factors not documented	6	3	2	2	0	3	1	1	0	0	2
MRSA, Methicillin-resistant Staphylococcus aureus										

**Table 4:** Distribution of the four most common predisposing risk factors in different age subgroups


**Age Group**	**Predisposing Risk Factor**	**Number of Patients**
< 18 years old	Contact lens wear	7
	Trauma	1
	Ocular surface disease	2
	Underlying corneal disease	–
18–40 years old	Contact lens wear	32
	Trauma	6
	Ocular surface disease	1
	Underlying corneal disease	2
> 40 years old	Contact lens wear	20
	Trauma	19
	Ocular surface disease	22
	Underlying corneal disease	30

**Table 5:** Rate of infectious keratitis in South Texas compared with other locations


	**% of culture-proven cases**	**% of bacterial cases from total positive cultures**	**% of fungal cases from total positive cultures**	**% of ** ***Acanthamoeba*** ** cases from total positive cultures**
South Texas	53.6%	95.1%	6.9%	1.6%
DEI-LA^[17]^	63%	89.8%	9.8%	0.4%
LAC + USC-LA^[17]^	82%	89.4%	10.6%	–
Dallas^[18]^	66%	85%	14.5%	0.5%
Miami^[19,20,21]^	40.1%	71.7%	20.8%	–
Mexico City^[22]^	37.6%	87%	13%	–
Bangladesh^[23]^	59%	21%	33%	–
South India^[24]^	70.6%	32.7%	34.4%	1%
DEI-LA Doheny Eye Institute – Los Angeles LAC + USC-LA Los Angeles County and University of Southern California Medical Center – Los Angeles			

**Table 6:** Percentage of bacterial isolates in South Texas compared with other locations


	*CoNS*	*Pseudomonas aeruginosa*	*Staphylococcus aureus*	*Moraxella*	*Streptococcus pneumoniae*
South Texas	25.7%	23.4%	11%	7.8%	2.8%
DEI-LA^[17]^	44.9%	13.1%	9.8%	- 1.9%
LAC+USC-LA^[17]^	24.6%	7.6%	8.8%	1.8%	1.8%
Dallas^[18]^	19.2%	18.7%	9.6%	4.2%	6.9%
Miami^[21]^	1.3%	25.7%	19.4%	0.8%	-
Mexico City^[22]^	39.3%	13.4%	21.7%	1.5%	2.8%
Bangladesh^[23]^	32.9%	20%	26.2%	- 8.6%
South India^[24]^	18.2%	19.9%	3.6%	0.8%	36%
DEI-LA Doheny Eye Institute- Los Angeles LAC+ USC-LA Los Angeles County and University of Southern California Medical Center – Los Angeles				

**Table 7:** Percentage of fungal species retrieved from the total fungal-positive cultures in South Texas compared with other locations


	*Fusarium*	*Aspergillus*	*Candida spp.*
South Texas	46.2%	–	7.7%
DEI-LA^[17]^	9.1%	13.6%	50%
LAC + USC-LA^[17]^	8.3%	12.5%	37.5%
Dallas^[18]^	28.1%	12.5%	15.6%
Miami^[20]^	54.1%	17.3%	26.2%
Mexico City^[22]^	50%	19.4%	1.4%
Bangladesh^[23]^	26.3	50.5%	–
South India^[24]^	41.9%	25%	–
DEI-LA Doheny Eye Institute – Los Angeles LAC + USC-LA Los Angeles County and University of Southern California Medical Center – Los Angeles		

### Fungal Keratitis

The six fungal species cultured in South Texas included *Fusarium* (n = 6, 46.2%), *Paecilomyces lilacinus* (*n* = 2, 15.4%), *Scedosporium* (*n* = 2, 15.4%), *Pleosporales *(*n* = 1, 7.7%), *Cladosporium* (*n* = 1, 7.7%), and *Candida parapsilosis* (*n* = 1, 7.7%). Fungal elements were noted on smears or confocal microscopy of six other patients, but fungal cultures were negative.

### Predisposing Risk Factors 

Contact lens wear was encountered in 59 (32.4%) of the 182 eyes with positive bacterial, fungal, or *Acanthamoeba* cultures. Other predisposing factors, which included chemical burns, graft failure, corneal dystrophies, epithelial defects, bullous keratopathy, and rosacea-induced keratitis, were identified in 32 eyes (17.6%) with culture-proven keratitis; 26 eyes (14.3%) were noted to have preceding trauma to the affected eye, and 25 (13.7%) were found to have ocular surface diseases, including significant dry eye disease. Thirty-seven patients were immunocompromised due to human immunodeficiency virus (HIV) infection, hepatitis, or the use of immunosuppressive drugs. Table 2 exhibits the predisposing conditions in this cohort. No risk factors were identified in 18 patients.

Table 3 shows the prevalent bacterial organisms found in patients with more common risk factors. The most common bacterial organism associated with contact lens use was *Pseudomonas aeruginosa* (*n* = 33, 57.9%), followed by CoNS (*n* = 16, 28.1%), and *Staphylococcus aureus* (*n* = 4, 7.0%). In patients with ocular surface disease and underlying corneal disease, however, CoNS was the most common bacterial organism, which was found in 13 eyes each (52.0% and 44.8%, respectively) in these two groups.

The observed risk factors in 13 patients with fungal keratitis included corneal diseases (*n* = 3, 23.0%), contact lens wear (*n* = 2, 15.4%), and preceding trauma (*n* = 2, 15.4%). Two cases of *Acanthamoeba* keratitis were associated with contact lens use (66.7%), whereas no predisposing factor was identified in the remaining *Acanthamoeba* case.

### Age Distribution in Different Causative Etiologies 

The average patient's age in the groups with bacterial and fungal keratitis was 48.2 ± 21.0 years and 52.1 ± 18.9 years, respectively. The average patients' age in the group with *Acanthamoeba *keratitis was 32.0 ± 6.4 years which was lower than that in the other two groups. The majority of bacterial and fungal keratitis occurred in patients > 40 years old (63.4% and 76.9%, respectively), followed by patients aged from 18 to 40 years (30.2% and 23.1%, respectively) and patients < 18 years old (6.4% and 0%, respectively). A single case of *Acanthamoeba* keratitis was identified in each age group.

### Prevalence of Risk Factors According to Age Group

The mean age of the subgroup with contact lens-associated keratitis was 35.8 ± 16.1 years, which was less than that of the subgroups with infectious keratitis caused by trauma (47.3 ± 16.1 years), ocular surface disease (57.5 ± 19.4 year), and underlying corneal diseases (63.8 ± 18.3 years). The majority of patients with contact lens-associated keratitis occurred in patients aged from 18 to 40 years (54.2%), followed by patients > 40 years old (33.9%) and those < 18 years old (11.9%). Underlying corneal disease (93.8%), preceding trauma (73.1%), and ocular surface disease (33.9%) were significantly more common in patients > 40 years old as compared to the patients < 40 years old. Table 4 summarizes the number of patients in each age subgroup with the four most common predisposing risk factors for corneal ulcer.

### Seasonal Variation

The majority of cases with corneal ulcer (*n* = 104, 57.1%) presented between October and March, during which the average temperature is lowest in South Texas, and the remaining cases presented between April and September (*n* = 78, 42.9%). The inclination toward lower temperatures was observed in the bacterial (*n* = 100, 58.1%) and *Acanthamoeba* (*n* = 3, 100%) corneal ulcer.

**Figure 1:**
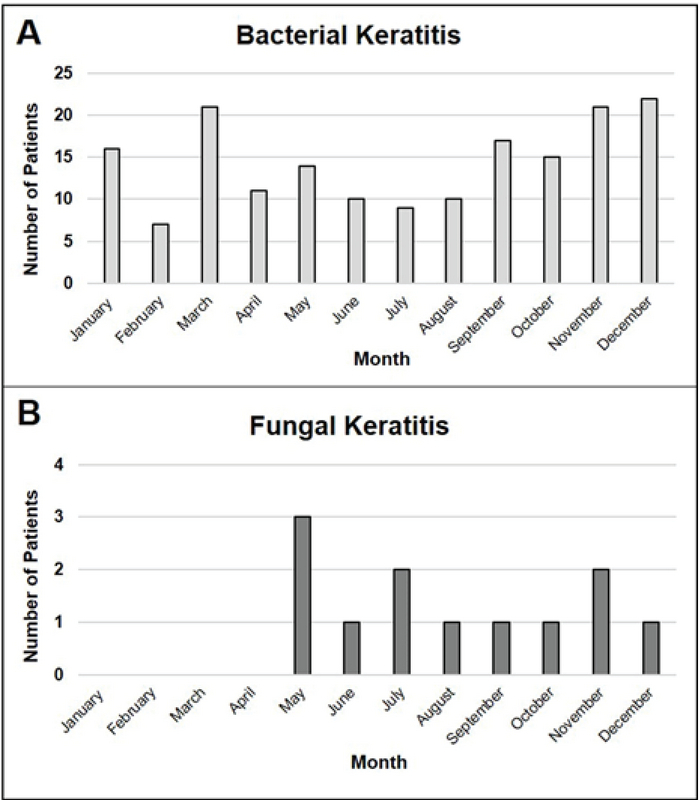
Seasonal distribution of occurrence of bacterial (A) and fungal (B) keratitis in South Texas.

The occurrence of bacterial keratitis had a bimodal distribution, which included a peak in March and another in November–December [Figure 1(A)]. The majority of cases with CoNS- (58.9%) and *Moraxella *(76.5%)-induced keratitis occurred in the colder months. No specific seasonal pattern was observed for the other bacterial cases. Approximately two-thirds of the fungal corneal ulcers occurred during the warmer months [Figure 1(B)]. All three cases of *Acanthamoeba* keratitis occurred in November and December 2014.

##  DISCUSSION

In our study, of the 621 patients with a clinical diagnosis of keratitis, 181 patients had culture-proven keratitis, which was 53.6% of those for which cultures were obtained. The proportion of positive cultures acquired in this study was comparable with the reported ranges from studies in Los Angeles (California), Dallas (North Texas), Miami (Florida), Mexico, Bangladesh, and South India [Table 5].^[17][18][19,20,21][22][23][24]^ The sex distribution was virtually equal between females and males in our study, and the mean age of this patient population was similar to those reported in several other studies.^[12,17,18,22,23,25,26,27]^

In our study, bacterial keratitis was the most common type of infectious keratitis, followed by fungal and *Acanthamoeba* keratitis. This is consistent with several other studies conducted in Los Angeles, Dallas, Florida, and Mexico City, which have climate conditions comparable to South Texas [Table 5].^[17][18][19,20,21][22]^ However, we found fewer cases of fungal keratitis compared with these studies. To identify the causative organisms, we only used cultures because smears typically cannot be used to diagnose the type of organism, especially in bacterial and fungal infections. This might have contributed to fewer observed cases of fungal keratitis in our study, since cultures for the diagnosis of fungal keratitis are not as sensitive as other forms of diagnostic techniques.^[28]^ In addition, although certain specialized stains, such as acridine , calcofluor white, and lactophenol-cotton blue, can be used to detect *Acanthamoeba* in smears, such stains were not used in our study, and *Acanthamoeba* keratitis was diagnosed only based on positive cultures. Furthermore, although *in vivo* confocal microscopy can be used to detect *Acanthamoeba* and fungi in the cornea,^[29]^ we did not use this method as it is not available in many facilities.

Florida has a notably higher rate of fungal keratitis (20.8%) than South Texas and several other locations in North America with similar latitudes [Table 5]. This higher rate is likely due to the tropical climate in Florida; this finding is further supported by the results of other studies that reported a high rate of fungal keratitis in tropical climate regions, such as Bangladesh and South India.^[19]^ In developing countries, however, this may be confounded by the large percentage of patients with fungal keratitis related to agricultural work.^[19,23,24]^

The predominant bacterial isolate was CoNS, which is consistent with the findings from Dallas, Los Angeles, Mexico City, and Bangladesh [Table 6].^[17][18][19,20,21][22][23]^ CoNS has consistently been shown to be a common cause of bacterial keratitis as it inhabits the skin and can invade a compromised cornea.^[23]^ Our study further supports this concept, as CoNS was the most common bacterial organism isolated in cases associated with underlying corneal and ocular surface diseases. Interestingly, the proportion of *Pseudomonas* keratitis, which was the second most common bacterial isolate, was higher in South Texas and Miami than the reported range of 7.6–20% found in other cities in the United States, Bangladesh, and South India [Table 6].^[17][18][19,20,21][22][23][24]^ The prevalence of *Moraxella* in South Texas (7.8%) and Dallas located in North Texas (4.2%) was higher than most other locations across the world.^[18]^ *Moraxella* is known to commonly affect immunocompromised patients, which is consistent with our cohort as some of our *Moraxella*-positive cases were immunocompromised individuals.^[30]^

*Fusarium*, a filamentous fungus, was found to be the predominant fungal isolate in our study, as well as in Dallas, Miami, Mexico City, and South India [Table 7].^[18,20,22,24]^ Interestingly, *Aspergillus*, which is typically associated with corneal trauma and tropical climates, was not isolated in South Texas; however, it has been isolated in several other studies with similar patient characteristics and climates.^[17,18,23,31]^ Filamentous fungi have been found to be more common than yeast fungi in areas with warmer climates, such as Texas, Florida, California, and Mexico.^[1]^ Our findings are consistent with this finding as only a single yeast fungus, *Candida*, was isolated in our study.

Several reports have found a positive association between filamentous fungal keratitis and contact lens use and ocular trauma,^[32]^ which may account for the more substantial proportion of filamentous fungal keratitis observed in our study as contact lens wear was the leading predisposing risk factor for infectious keratitis.

Contact lens wear was the most frequent risk factor associated with infectious keratitis in our study, followed by underlying corneal diseases, preceding ocular trauma, and ocular surface diseases. It is well established that the majority of cases with infectious keratitis in developed countries are related to contact lens use, while most cases in developing countries are caused by ocular trauma.^[14,18,23]^ In our study, contact lens-associated corneal ulcers were most prevalent in patients ≤ 40 years, whereas corneal ulcers associated with underlying corneal and ocular surface diseases were more frequently encountered in patients > 40 years old, which is similar to the results of previous studies.^[25,27,33]^ Furthermore, the occurrence of bacterial keratitis associated with contact lens use in our study is consistent with the range iterated by other studies in developed countries (31–53%).^[26,27,34,35]^ *Pseudomonas* has been reported as the most common bacterial species associated with contact lens wear in South Texas, Dallas, and Florida.^[18,21]^

In our study, approximately one-third of cases with fungal keratitis occurred in those with underlying corneal and ocular surface diseases. Fungal keratitis has often been attributed to trauma and contact lens wear.^[14,23,31]^ In a large multicenter study in the United States, 37% of fungal keratitis cases were associated with contact lens use, followed by ocular surface disease (29%) and ocular trauma (25%).^[32]^ However, this rate varies in different locations; for example, contact lens use was found to be the most common risk factor associated with fungal keratitis (41%) in Boston, but trauma was the most common risk for this type of infectious keratitis in Florida (44%).^[36,37]^

Despite many studies have reported an increase in infectious keratitis during the warmer months, the majority of our cases presented during the months with lower temperatures, showing a peak in March and November–December.^[15,27]^ CoNS and *Moraxella *were the only bacterial isolates to have a significant seasonal distribution, with the majority of cases occurring during the cooler months. Just over half of the CoNS cases associated with ocular surface disease were observed in the winter (53.8%). Ocular surface disease exacerbated by low temperatures may have put these patients at an increased risk of keratitis.^[38]^ As anticipated, the majority of cases with fungal keratitis occurred during the warmer months, which is consistent with the results of previous studies conducted in both developed and developing countries.^[15,39]^

In conclusion, bacteria were the most prevalent etiology of infectious corneal ulcers in South Texas. Coagulase-negative *Staphylococcus* and *Pseudomonas* were the most common bacterial isolates; this result is consistent with the results of other studies reporting the etiologies of bacterial keratitis in populations across the United States and the world. *Fusarium*, a filamentous fungus, was the most frequent fungal isolate, but the overall prevalence of fungal keratitis was lower in South Texas than in other cities of developed countries. As contact lens use is the most common risk factor associated with infectious keratitis in South Texas and many other populations, contact lens wearers should always be reminded of this potential sight-threatening complication.

##  Financial Support and Sponsorship

None.

##  Conflicts of Interest

There are no conflicts of interest.
